# A comparative study of the effectiveness of virtual reality-based and text-based training in sterile preparation: pilot trial of early-stage clinical VR study

**DOI:** 10.1186/s40780-026-00571-5

**Published:** 2026-04-15

**Authors:** Satoru Esumi, Sari Nakagawa, Mai Ikemura, Yui Takezawa, Rika Ebara, Miho Harada, Akitoshi Tatsumi, Akiko Kanmachi, Yasuhiko Hashimoto, Mariko Takeda-Morishita

**Affiliations:** Faculty of Pharmaceutical Sciences, Kobegakuin University, 1-1-3 Minatojima, Chuo-ku, Kobe, Hyogo, Japan

**Keywords:** Practical training, Virtual reality, Pharmacy students, Sterile preparation

## Abstract

**Background:**

This study is a pilot study evaluating the effectiveness of virtual reality (VR) applications in retraining sterile preparation techniques for injectable medications. Specifically, it focused on comparing VR-based learning with conventional text-based learning and objectively assessing students’ acquisition of practical skills using standardized evaluation criteria.

**Methods:**

Seventeen pharmacy students voluntarily participated in the study and were divided into two groups: one using a VR application for learning and the other using text-based materials. Immediately after training with their respective tools, students’ aseptic preparation skills were assessed using standardized criteria (including 29 individual evaluation checkpoints) and a 5-point overall rating scale.

**Results:**

Students who used the VR application completed tasks in a shorter time and scored higher on evaluation criteria such as operational smoothness and drug dissolution within vials. Additionally, multiple assessment items, including “Fill the aseptic smoothly and evenly, without stopping in the middle of the process,” were strongly correlated with the overall practical performance ratings of the VR group.

**Conclusions:**

The introduction of VR applications for aseptic preparation training, which involves complex procedures, suggests that VR-based learning may be more effective than traditional text-based methods in helping students understand procedural workflows and improve operational fluency. These findings indicate that VR technology may serve as a useful educational tool in practical pharmacy training. Further studies with larger sample sizes are needed to confirm these findings.

**Trial registration:**

Not applicable

## Background

Simulation-based learning experiences are widely used as one of the most effective training tools in education involving technology acquisition [1]. Simulation-based learning refers to the use of simulation environments and technologies for learning purposes, such as case-based learning, which provides learners with virtual learning experiences and realism [2]. More recently, three-dimensional technologies such as virtual reality (VR), augmented reality (AR), and mixed reality (MR) have been actively used in simulation-based learning [3]. Compared with conventional textbook-based learning, the introduction of such advanced technology into education is expected to reduce the time required for skill acquisition, the cost of teaching materials, and human resources.

Simulation-based learning using VR technology has already been incorporated and reported to be useful in other medical fields, such as medicine and nursing [4, 5]. However, although the development and introduction of VR in pharmacy education has lagged behind other medical fields, reports on its usefulness have increased in recent years [6, 7]. This type of hands-on training education using VR reportedly contributes to higher student satisfaction and motivation for learning than actual hospital training [8, 9]. However, the VR technologies described in these reports appear to employ passive educational approaches, such as viewing content through 360-degree videos, rather than active learning methods that require student interaction and engagement. In addition, previous studies have primarily evaluated the effects of VR on pharmacy education on the basis of surveys of students’ knowledge improvement and subjective satisfaction. A limited number of studies have evaluated the effectiveness of VR-based instructional materials that involve active learner engagement, in comparison to traditional text-based learning.

We originally developed a VR application that enables pharmacy students to acquire a broad range of knowledge and skills related to dispensing techniques and patient consultation. Using this application, we initiated practical pharmacy education. Previous research has demonstrated that education using this VR application achieves high student satisfaction and is particularly effective for learning the dispensing techniques of oral liquid formulations [10]. However, the effectiveness of the VR application for acquiring more complex dispensing techniques has not yet been fully examined. Therefore, this study evaluated the effectiveness of a VR application for teaching aseptic preparation techniques for injectable drugs, which require advanced procedures. Specifically, this study aimed to determine whether the VR application could support the acquisition of complex procedural skills, such as the handling of needles and glass containers. To minimize the risk of injury, a pilot study was conducted with a small number of students who had previously completed practical training.

In Japanese pharmacy education, standardized tests are used to assess practical skills. Accordingly, this study also employed standardized scales to objectively evaluate students’ acquired skills. Specifically, the time required for each student to complete all procedures and the achievement status of each evaluation checkpoint were recorded. Furthermore, students’ overall performance across the aseptic preparation task was evaluated using a five-point comprehensive assessment score, and a two-dimensional plot was constructed to identify which checkpoint items positively contributed to the comprehensive assessment score. This analysis was intended to enable a more detailed investigation of the educational effectiveness of each instructional method and to clarify which elements of the procedural steps functioned effectively or ineffectively. By introducing such an evaluation method, we believe that it will be possible to measure educational effectiveness with greater accuracy compared with subjective survey-based assessments. Furthermore, if the results of this study confirm that use of the VR application facilitates the acquisition of advanced skills in a short period, it is expected to contribute to the development of a new simulation-based learning method in practical pharmacy education.

## Methods

### Study design and participants

The study recruited fourth-year students from the Faculty of Pharmaceutical Sciences, Kobe Gakuin University (Kobe, Japan), who participated on a voluntary basis; a total of 17 students were enrolled. Because injectable drug preparation involves potentially hazardous procedures, such as needle-stick injuries, the study was conducted after all participants had completed one prior hands-on training session using actual injectable drugs. Specifically, participants had previously undertaken four practical training sessions, consisting of two vial-based and two ampule-based preparations. Upon completion of the prior hands-on training session, all students had reached a level enabling them to perform aseptic procedures while following the instructor’s instructions. This study therefore aimed to evaluate the effects of VR-based or text-based learning on the relearning and recollection of aseptic preparation procedures.

Aseptic training in this study was conducted according to the following schedule. First, an introductory lecture on aseptic preparation was provided, followed by individual learning through instructional videos. The content of the introductory lecture and instructional videos comprehensively covered all evaluation items and procedural steps assessed in the practical examination. Students then participated in group-based hands-on training using actual equipment and pharmaceutical materials. The training session lasted approximately 3.5 hours and consisted of a lecture of up to 30 minutes, followed by approximately 3 hours of hands-on practice. After observing an instructor-led demonstration, students performed the aseptic preparation procedure themselves. Training was conducted in groups of four students, with two students performing the procedure simultaneously while the other two observed and alternated throughout the session. Consequently, each student had approximately 90 minutes of active hands-on practice. One instructor was assigned to every four students, and faculty supervision was provided throughout the session. After an interval of approximately 2–3 months, a retraining session using a VR application or text-based materials and an assessment test were conducted. Between the initial hands-on training and the retraining session, students were allowed to independently review the instructional videos and lecture materials, except during the retraining and assessment test.

### Learning tools: VR application and text-based material

The VR application used in this study was created by ImaCreate Co. Ltd. under supervision of the authors and was serviced by Fujifilm System Service Corporation (Pharmacy Pre-Learning VR Training Service; Tokyo, Japan). In the VR application, each subsequent necessary task is shown step-by-step, and the student proceeds to the next step when he or she performs the necessary operation. The VR application was provided via all-in-one VR headsets (Meta Quest 2®). Compared to the VR material, the text material provides only a detailed textual description of the standard aseptic preparation process, is developed independently by the authors from textbooks, and lacks any visual content. Both the VR application and the text material ensured that all checkpoint items were covered in the learning process.

### Aseptic preparation task

The same aseptic preparation task was used for both the learning session and the practical test. The injectable drugs used included a vial of VITAMEDIN® for intravenous injection (Alfresa Pharma Corp.), 20 mL plastic ampules of distilled water for injection (Otsuka Pharmaceutical Factory, Inc.), and a 100 mL bag of isotonic sodium chloride solution for injection (Terumo Corp.). The standard procedure was as follows:

1. 5 mL of distilled water was aspirated from the ampule into a syringe.
2. Using a negative pressure maneuver, the 5 mL of water was injected into a vial of VITAMEDIN® to dissolve its contents.
3. The entire volume of the dissolved solution was then aspirated back into the syringe and injected into the 100 mL saline bag.
4. The solution in the saline bag was gently mixed by inversion and visually inspected for particulate matter.

This task served as the basis for both the instructional learning and the assessment of aseptic technique in this study.

### Evaluation schedule

The participants were randomly divided into two groups: one for learning with the VR application (VR group) and the other for learning with textbook-based materials (Tx group). Each group was given 30 minutes to study the aseptic preparation task described above.

The evaluation checkpoints consisted of 29 items (Table 1), assessed according to Japan’s Objective Structured Clinical Examination (OSCE), a standardized practical assessment administered to fourth-year pharmacy students prior to their clinical training. For checklist item 4 (correct order of the aseptic filling process), a single correct procedural sequence was predefined based on OSCE criteria and standard aseptic preparation guidelines. The checklist items numbered 7 through 29 were arranged in a sequence reflecting appropriate sterile workflow and contamination control. Students were instructed consistently during prior hands-on training as well as in both the VR and text-based materials to perform these steps in the prescribed top-to-bottom order. For the checklist item assessing “correct order,” adherence to this predefined sequence was evaluated, and deviations from the prescribed order were classified as incorrect. For checklist item No. 13 (inserting the needle vertically into the vial without approaching from above), top-down needle insertion was classified as incorrect. Although vertical insertion from above may not necessarily be inappropriate if the plunger is securely stabilized, habitual top-down insertion may increase the risk of unintended dripping from the needle tip if the plunger is not adequately fixed, particularly when handling hazardous drugs. In addition, during repeated vial punctures, inserting the needle while visually confirming the puncture site may reduce the risk of coring caused by repeated insertion at the same location. For these safety and technical reasons, students at our institution are instructed to avoid top-down needle insertion as part of standard aseptic technique training. For the checklist item assessing drug dissolution in a vial (Item No. 15), adherence to a predefined safe dissolution technique was required for the item to be scored as achieved. This technique required maintaining the syringe inserted into the vial and holding both the vial and syringe in the prescribed position during mixing. Removal of the needle prior to dissolution was classified as incorrect for this item, even if the full volume was subsequently recovered.

**Table 1:** Checkpoints for aseptic preparation tasks in this study

No.	Checkpoints	Successful subjects, n(%)		P value
		VR group (*n* = 9)	Tx group (*n* = 8)	
1	Do not allow the needle tip to come into contact with anything.	8 (88.9)	5 (62.5)	0.294
2	Handle the alcohol swab cleanly with tweezers and properly disinfect the puncture site.	9 (100)	6 (75)	0.206
3	Do not touch the disinfected area, especially areas that have been wiped with alcohol.	9 (100)	8 (100)	1
4	Perform the aseptic filling process in the correct order.	4 (44.4)	3 (37.5)	1
5	Fill the aseptic smoothly and evenly, without stopping in the middle of the process.	7 (77.8)	1 (12.5)	0.015
6	Fill the aseptic in the correct position on a clean bench (guideline: perform the operation at least 15 cm in from the edge of a clean bench).	9 (100)	8 (100)	1
7	Confirm that the prescription and items match (via pointing or by line of sight).	9 (100)	6 (75)	0.206
8	Wipe the rubber stopper of the bag with alcohol.	9 (100)	8 (100)	1
9	Turn the opening of the bag toward the back so that your hands do not touch the opening of the bag during aseptic filling.	8 (88.9)	8 (100)	1
10	Wipe the rubber stopper of the vial with an alcohol cotton swab.	9 (100)	8 (100)	1
11	Wipe the lip of the plastic ampule with a new alcohol cotton swab.	9 (100)	6 (75)	0.206
12	Confirm that the correct amount of water for injection (5 mL) has been retrieved (via pointing or by line of sight).	8 (88.9)	6 (75)	0.576
13	Insert the vertically held needle into the rubber stopper (do not insert the needle from above).	5 (55.6)	2 (25)	0.335
14	Inject the full volume of solution via negative pressure (keep the vial on the table with the syringe inserted into the vial, then pull the syringe plunger straight up).	8 (88.9)	4 (50)	0.131
15	Dissolve the drug in the vial (with the syringe inserted into the vial, hold the vial on top and the syringe on the bottom, then retain this positioning and shake the vial and syringe with both hands).	5 (55.6)	0 (0)	0.029
16	Confirm that the drug in the vial is dissolved (via pointing or by line of sight).	4 (44.4)	4 (50)	1
17	Retrieve the drug in the vial using negative pressure (with the syringe inserted into the vial, the vial on top, and the syringe on the bottom, pull the syringe plunger straight down).	8 (88.9)	5 (62.5)	0.294
18	Check if the vial is empty.	4 (44.4)	6 (75)	0.335
19	Discard the vial.	6 (66.7)	8 (100)	0.206
20	Remove any air from inside the syringe	8 (88.9)	6 (75)	0.576
21	Check the amount of drug retrieved in the syringe (by pointing or via line of sight).	8 (88.9)	6 (75)	0.576
22	Insert the needle vertically into the bag (do not insert the needle from above)	8 (88.9)	5 (62.5)	0.294
23	Inject the full volume of the drug solution into the bag.	9 (100)	8 (100)	1
24	Discard the needle in the sharps disposal box (do not recap it).	7 (77.8)	8 (100)	0.471
25	Wipe the opening of the bag with alcohol.	8 (88.9)	7 (87.5)	1
26	Wipe the cap with an alcohol cotton swab.	9 (100)	6 (75)	0.206
27	Place the cap on the mouth of the bag.	9(100)	7 (87.5)	0.471
28	Tilt and mix the bag (up and down, left and right).	9 (100)	6 (75)	0.206
29	Check both the front and back sides of the bag for foreign matter.	9 (100)	6 (75)	0.206

Checkpoints for aseptic preparation tasks for injectable drugs and differences in achievement between VR and Tx groups. Data were analyzed by the Fisher’s exact probability test. VR group: Students who studied with the VR tool (*n* = 9). Tx group: Students who used text-based learning (*n* = 8).

In addition, overall performance was evaluated using a 5-point scale based on OSCE criteria (5 = Excellent performance with no errors; 4 = Good performance with minor errors; 3 = Acceptable performance with moderate errors; 2 = Poor performance with multiple errors; 1 = Unacceptable performance). The time required for aseptic preparation was measured and censored at 11 minutes.

To reduce inter-rater variability in this small-scale pilot study, all procedures were video-recorded and evaluated by a single assessor. The assessor had extensive experience in hospital-based aseptic preparation and more than 15 years of experience in OSCE-based sterile preparation instruction, ensuring substantial expertise in performance evaluation. To minimize potential assessment bias, the assessor was blinded to group allocation.

A single video camera was positioned at a fixed location in front of the participant to ensure that the entire working area and the participant were within the frame, with continuous visibility of hand movements. In a preliminary assessment, we confirmed that this camera position allowed reliable evaluation of detailed procedural steps from the recorded videos. The camera had a resolution of approximately 8.3 megapixels, and image magnification enabled sufficient visualization of fine details, including the needle tip.

### Safety monitoring of participants

Following completion of the task, participants were orally queried regarding any adverse symptoms during VR use, including dizziness, nausea, or eye strain. Any physical discomfort reported by participants in either group during the study was documented, and appropriate measures were taken as necessary to ensure participant safety.

### Data analyses

The time required for preparation was compared using the Kaplan–Meier method (log-rank test). The comparison of groups’ achievement of each checkpoint item was analyzed using Fisher’s exact probability test, and the comprehensive evaluation throughout the aseptic preparation task was compared using the Mann‒Whitney U test. To identify which components of each instructional method were effective or ineffective, and to evaluate which checkpoint items had a positive impact on comprehensive evaluation scores within each instructional method, a two-dimensional plot was constructed using two variables [1]: the standardized score (with a mean of 50 and a standard deviation of 10) of Spearman’s rank correlation coefficient between each checkpoint’s achievement rate and the comprehensive evaluation score, and [2] the standardized score (with a mean of 50 and a standard deviation of 10) of the achievement rate for each checkpoint. Similar analytical methods have also been applied in the evaluation of pharmacy education, supporting their utility in assessing instructional approaches and identifying areas for improvement [11].

All the statistical analyses were performed with Easy R (Saitama Medical Center, Jichi Medical University, Saitama, Japan), which is a graphical user interface for R (The R Foundation for Statistical Computing, Vienna, Austria). More precisely, it is a modified version of the R commander designed to add statistical functions frequently used in biostatistics [12]. The statistical significance level was set at 5%, and results with *p* < 0.1 were considered to indicate a trend.

### Ethical considerations

This study was approved by the Kobe Gakuin University Human Research Ethics Committee (SEB23-01). All participants were informed of the study in writing, and written informed consent was obtained. All procedures involving human participants were performed in accordance with the ethical standards of institutional and national research committees and the 1964 Helsinki Declaration.

## Results

### Effect of learning aseptic preparation using the VR application on the time required for the practical examination and completion rates

**Fig. 1:**
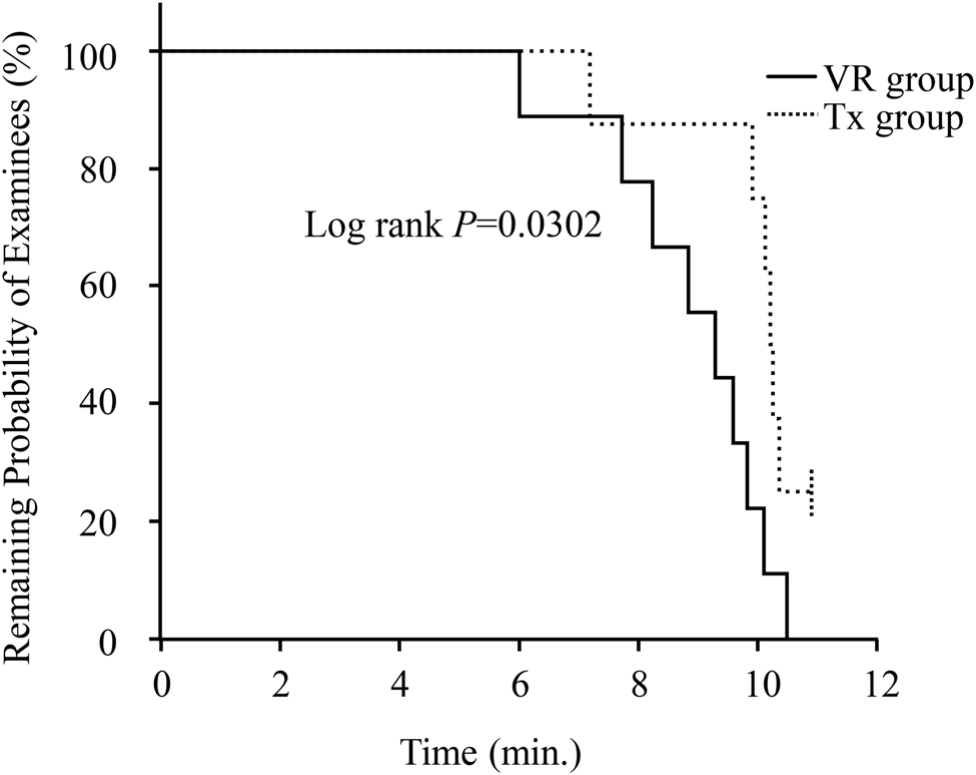
Kaplan–Meier curves for time to complete assessment tasks for different learning method. The solid line shows the residual rate over time for students who studied with the VR application (VR group, *n* = 9), and the dashed line shows the residual rate over time for students who studied with the text (Tx group, *n* = 8). The residual rate decreases as each group of students finishes their task. Data were compared using the Kaplan-Meier method followed by the log-rank test. The significance level was set at *p* < 0.05

The number of students who completed the injection preparation within the time limit (n, %) was 9/9 (100%) in the VR group and 6/8 (75%) in the Tx group. Kaplan–Meier curves comparing the residual probability of students completing the practical tasks over time for the VR group and the Tx group are shown in Fig. 1. The log-rank test results indicated that students in the VR group completed the assignment more expeditiously. The median completion time was 9.3 minutes for the VR group and 10.2 minutes for the Tx group (*p* < 0.05).

### Impact of differences in learning materials on the evaluation of practical tests

Table 1 presents the evaluation items used in the practical test, along with the achievement ratios (i.e., the percentage of students who indicated they had completed each item). When a student did not complete the procedure within the allotted time, any unperformed checklist items were counted as not achieved. Among the two students in the Tx group who did not complete the procedure, one failed to complete items 28 and 29 (Table 1) due to time expiration, and the other failed to complete items 25–29 (Table 1) for the same reason. Compared with the Tx group, the VR group demonstrated consistently greater achievement in two items: “Fill the aseptic smoothly and evenly, without stopping in the middle of the process” (*p* = 0.015), and “Dissolve the drug in the vial (with the syringe inserted into the vial, hold the vial on top and the syringe on the bottom, then retain this positioning and shake with both hands)” (*p* = 0.029). Additionally, the mean number of checkpoint items achieved was 24.7 ± 3.5 in the VR group and 20.9 ± 4.7 in the Tx group (*p* = 0.09), suggesting a potential trend toward greater achievement in the VR group.

**Fig. 2:**
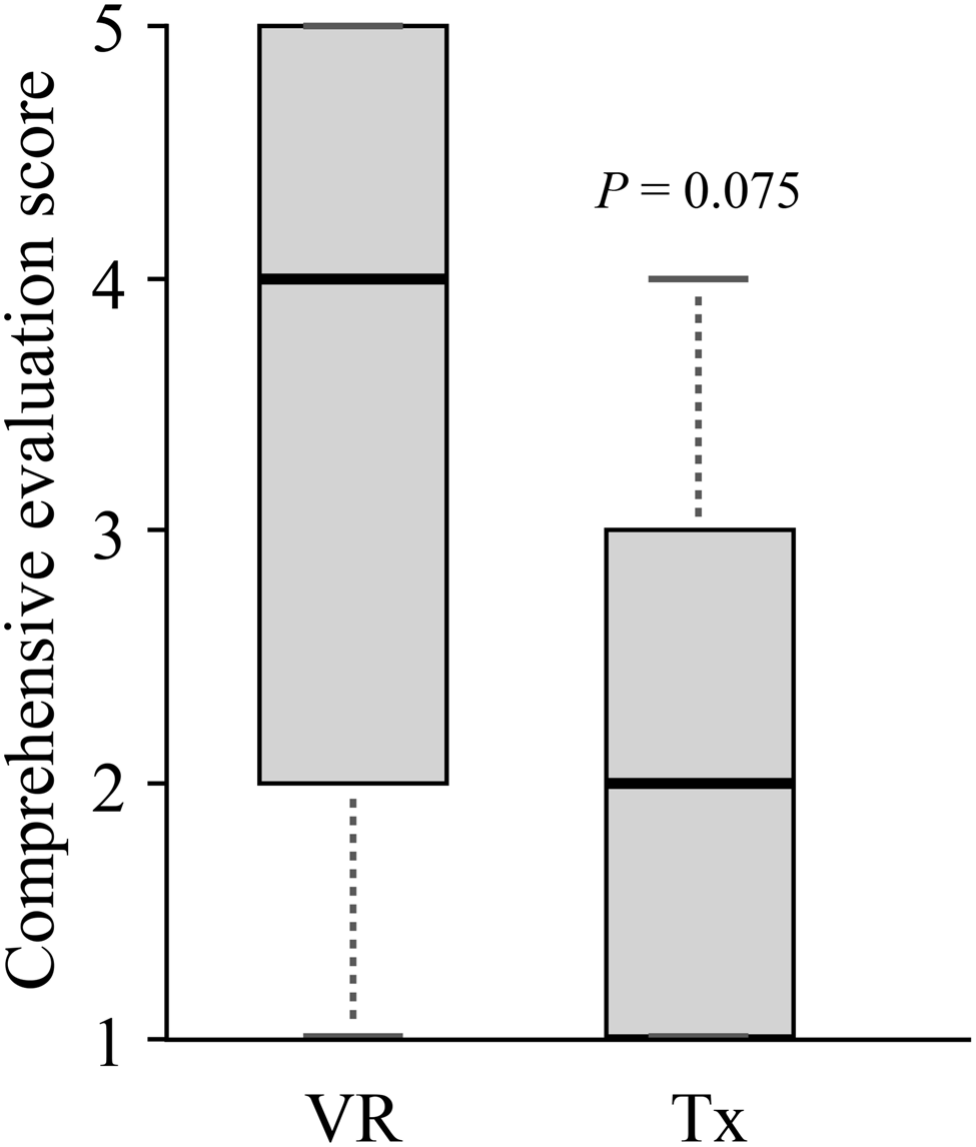
The influence of different learning methods on the comprehensive evaluation. The degree of completion of the technique throughout the task was rated on a 5-point scale for each student and is shown in a histogram by group. Data were analyzed with the Mann-Whitney U test. The significance level was set at *p* < 0.05, and results with *p* < 0.1 were considered to indicate a trend

The median (minimum–maximum) comprehensive evaluation score for the practical test of aseptic preparation of injectable drugs, assessed on a 5-point scale, was 4 [1–5] for the VR group and 2 [1–4] for the Tx group, indicating a higher score trend in the VR group (*p* = 0.075; Fig. 2).

### Relationship between comprehensive evaluation and achievement ratio of each checkpoint

**Fig. 3:**
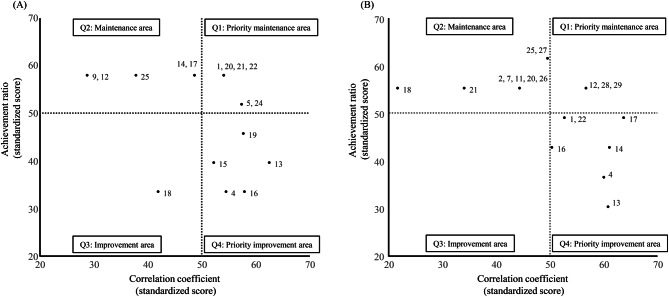
Two-dimensional plot of checkpoint achievement and correlation with comprehensive evaluation. Figure **A** shows the VR group, and Figure **B** the Tx (text-based learning) group. Each panel displays a two-dimensional plot based on two axes: the standardized achievement ratio for each checkpoint (y-axis) and the standardized Spearman’s rank correlation coefficient between the comprehensive evaluation score and each checkpoint achievement (x-axis). The numerical labels(e.g., 1, 2, …)indicate checkpoint numbers corresponding to those listed in Table 1. Checkpoints in the upper right quadrant (Q1) demonstrate both high achievement rates and strong positive correlations with the comprehensive evaluation, suggesting these steps contribute positively to overall performance and should be prioritized for maintenance. In contrast, those in the lower right quadrant (Q4) exhibit low achievement rates but high correlation with overall performance, indicating priority improvement areas—students who failed these steps often received low overall ratings

A two-dimensional plot based on the correlation coefficient (standardized score) between the comprehensive evaluation score and each checkpoint, and the achievement ratio (standardized score) for each checkpoint are shown in Fig. 3. The numerical labels attached to each plot indicate the checkpoint numbers referenced in Table 1. The checkpoints in the first quadrant (upper right-hand corner) of the graph had a positive impact on the comprehensive evaluation due to the high achievement ratio for that item and the high correlation coefficient with the comprehensive evaluation. On the other hand, the checkpoints in the fourth quadrant (lower right-hand corner) showed a negative impact on the comprehensive evaluation due to the low achievement ratio for that item itself and its strong association with the comprehensive evaluation. Achievement ratio for each checkpoint and the Spearman’s correlation coefficient between the comprehensive evaluation score and the achievement ratio are summarized in Table 2 for clarity.

**Table 2:** Spearman’s ρ and achievement ratios for each checkpoint item in VR and Tx groups

No.	Checkpoints	VR		Tx	
		Achievement ratio	Spearman’s ρ	Achievement ratio	Spearman’s ρ
1	Do not allow the needle tip to come into contact with anything.	0.889	0.562	0.625	0.537
2	Handle the alcohol swab cleanly with tweezers and properly disinfect the puncture site.	－	－	0.750	0.267
3	Do not touch the disinfected area, especially areas that have been wiped with alcohol.	－	－	－	－
4	Perform the aseptic filling process in the correct order.	0.444	0.578	0.375	0.775
5	Fill the aseptic smoothly and evenly, without stopping in the middle of the process.	0.778	0.690	0.125	0.524
6	Fill the aseptic in the correct position on a clean bench (guideline: perform the operation at least 15 cm in from the edge of a clean bench).	－	－	－	－
7	Confirm that the prescription and items match (via pointing or by line of sight).	－	－	0.750	0.267
8	Wipe the rubber stopper of the bag with alcohol.	－	－	－	－
9	Turn the opening of the bag toward the back so that your hands do not touch the opening of the bag during aseptic filling.	0.889	−0.421	－	－
10	Wipe the rubber stopper of the vial with an alcohol cotton swab.	－	－	－	－
11	Wipe the lip of the plastic ampule with a new alcohol cotton swab.	－	－	0.750	0.267
12	Confirm that the correct amount of water for injection (5 mL) has been retrieved (via pointing or by line of sight).	0.889	−0.421	0.750	0.667
13	Insert the vertically held needle into the rubber stopper (do not insert the needle from above).	0.556	0.889	0.250	0.800
14	Inject the full volume of solution via negative pressure (keep the vial on the table with the syringe inserted into the vial, then pull the syringe plunger straight up).	0.889	0.351	0.500	0.808
15	Dissolve the drug in the vial (with the syringe inserted into the vial, hold the vial on top and the syringe on the bottom, then retain this positioning and shake the vial and syringe with both hands).	0.556	0.489	－	－
16	Confirm that the drug in the vial is dissolved (by pointing or visual confirmation).	0.444	0.711	0.500	0.462
17	Retrieve the drug in the vial using negative pressure (with the syringe inserted into the vial, the vial on top, and the syringe on the bottom, pull the syringe plunger straight down).	0.889	0.351	0.625	0.894
18	Check if the vial is empty.	0.444	0.089	0.750	−0.467
19	Discard the vial.	0.667	0.702	－	－
20	Remove any air from inside the syringe	0.889	0.562	0.750	0.267
21	Check the amount of drug retrieved in the syringe (by pointing or visual confirmation).	0.889	0.562	0.750	−0.067
22	Insert the needle vertically into the bag (do not insert the needle from above)	0.889	0.562	0.625	0.537
23	Inject the full volume of the drug solution into the bag.	－	－	－	－
24	Discard the needle in the sharps disposal box (do not recap it).	0.778	0.690	－	－
25	Wipe the opening of the bag with alcohol.	0.889	−0.070	0.875	0.436
26	Wipe the cap with an alcohol cotton swab.	－	－	0.750	0.267
27	Place the cap on the mouth of the bag.	－	－	0.875	0.436
28	Tilt and mix the bag (up and down, left and right).	－	－	0.750	0.667
29	Check both the front and back sides of the bag for foreign matter.	－	－	0.750	0.667

Spearman’s correlation coefficients (Spearman’s ρ) and achievement ratios for each checkpoint item in VR and Tx groups. When the achievement ratio is 1 or 0, Spearman’s ρ cannot be calculated, so they were omitted (–).

The numbers next to each plot correspond to the checkpoint numbers listed in Table 1. Checkpoints with an achievement ratio of 1 or 0 were excluded, as correlation coefficients could not be calculated for these items. In both the VR and text-based (Tx) groups, several checkpoints were commonly located in the fourth quadrant of the graph, indicating that lower achievement on these items was associated with lower comprehensive evaluation scores. Specifically, checkpoint 4 (performing the aseptic filling process in the correct order), checkpoint 13 (inserting the vertically held needle into the rubber stopper without approaching from above), and checkpoint 16 (confirming that the drug in the vial is dissolved, either by pointing or visual inspection) were situated in this quadrant.

In addition to these shared checkpoints, the VR group exhibited two items uniquely located in the fourth quadrant: checkpoint 15 (dissolving the drug in the vial with the syringe inserted, by holding the vial on top and the syringe on the bottom, and shaking them with both hands while maintaining this position), and checkpoint 19 (discarding the vial). In contrast, four items were uniquely located in the fourth quadrant of the Tx group plot: checkpoint 1 (ensuring that the needle tip does not come into contact with any surface), checkpoint 14 (injecting the full volume of solution via negative pressure), checkpoint 17 (retrieving the drug using negative pressure with the vial on top and the syringe on the bottom, pulling the plunger straight down), and checkpoint 22 (inserting the needle vertically into the bag, avoiding top-down insertion).

Checkpoints located in the first quadrant—those with both high achievement rates and strong positive correlations with comprehensive evaluation scores—differed notably between the VR and Tx groups. This suggests that the checkpoint items contributing to improved performance varied depending on the learning material. In the VR group, higher comprehensive scores were associated with successful completion of checkpoint 1 (avoiding contact of the needle tip with any surface), checkpoint 5 (smooth and continuous filling), checkpoint 20 (removing air from inside the syringe), checkpoint 21 (checking the volume of the drug drawn into the syringe), checkpoint 22 (inserting the needle vertically into the bag), and checkpoint 24 (disposing of the needle without recapping it). In the Tx group, on the other hand, higher comprehensive evaluation scores were associated with successful completion of checkpoint 12 (confirming that the correct amount of water for injection [5 mL] has been retrieved, either by pointing or visual inspection), checkpoint 28 (tilting and mixing the bag up and down, left and right), and checkpoint 29 (checking both the front and back sides of the bag for foreign matter).

Regarding safety outcomes, no severe cybersickness or VR-related adverse events requiring discontinuation of the examination were observed. In addition, no participants in either group reported any physical discomfort during the study period.

## Discussion

This study examined the usefulness of VR educational applications as learning materials for the aseptic preparation of injectable drugs, a technique that pharmacy students should master. Students who learned using the VR application completed the task in a shorter time during the evaluation test. The VR group also achieved greater scores on two items of the checkpoint, while no differences were observed for the remaining items. Furthermore, both the comprehensive evaluation scores and the average number of checkpoint items achieved tended to be higher in the VR group. Previous studies examining the usefulness of VR in education have relied primarily on subjective impressions [8, 13, 14]. In contrast, our study objectively assessed the acquisition of practical skills, representing a novel approach in the field of pharmacy education. Our findings resemble those reported in surgical training research, where VR-based learning was associated with increased procedural accuracy, reduced task duration and error rates, and increased learner confidence [15]. To our knowledge, this study is the first in pharmacy education to demonstrate, through objective metrics, the effectiveness of VR in supporting the development of procedural skills.

The VR group completed a greater number of checkpoint items than the Tx group, suggesting that the shorter task completion time observed in the VR group was not due to the omission of procedural steps, but rather reflected improved fluency in operational execution. This interpretation is supported by the higher achievement rate for the item “Fill the aseptic smoothly and evenly, without stopping in the middle of the process” in the VR group. A similarly high achievement rate was observed for the item “Dissolve the drug in the vial (with the syringe inserted into the vial, hold the vial on top and the syringe on the bottom, then retain this positioning and shake the vial and syringe with both hands).” The capacity to visualize spatial relationships and procedural techniques within the VR environment may have contributed to the successful performance of these steps. These higher individual item scores, along with the shorter task completion time, were also reflected in the comprehensive evaluation results. Among the two students in the Tx group who did not complete the procedure, one left items 28 and 29 unfinished due to time expiration, and the other left items 25–29 unfinished. Accordingly, the low achievement rates observed for certain items in Table 1 (e.g., Items 4, 15, and 16) are unlikely to be attributable to incomplete procedures due to time constraints. Rather, these items were completed but scored as not achieved due to procedural errors. The low implementation rates observed for the dissolution technique (approximately 50% in the VR group and 0% in the Tx group) warrant careful interpretation. In this study, correct performance required maintaining the syringe inserted into the vial and stabilizing both the vial and syringe in the prescribed position during mixing to ensure safe handling and maintenance of aseptic integrity. Therefore, even if students were able to subsequently recover the full volume, deviation from this predefined safe handling technique was classified as incorrect.

The primary reason for non-achievement of this dissolution-related checklist item was improper stabilization and hand positioning during dissolution. Although students had previously learned the correct technique during hands-on training and had access to illustrated instructional materials, differences in immediate recall associated with the review method (VR vs. text-based learning) appeared to influence performance. Text-based materials may be less effective in conveying dynamic hand positioning and spatial relationships, whereas VR provides visual and procedural guidance. Nevertheless, the fact that this item remained challenging even in the VR group suggests that dissolution technique is a complex procedural skill that may require more focused and repeated training.

The two-dimensional plot evaluating the relationship between the achievement ratio and the correlation coefficient with the comprehensive evaluation score revealed distinct patterns across the VR and Tx groups. To interpret the educational significance of each quadrant, we considered the distribution of items based on achievement levels and their associations with overall performance [11]. The items in the first quadrant, which exhibited both high achievement and strong correlations with the comprehensive evaluation scores, were regarded as priority maintenance areas. These represent critical steps that contribute meaningfully to student performance and should be preserved through consistent instructional reinforcement. Conversely, items in the second quadrant, which also had high achievement but lower correlations with overall evaluation, were interpreted as general maintenance areas—indicating steps that students performed well but that had relatively limited influence on final performance. The items in the third quadrant, characterized by low achievement and weak association with overall performance, were classified as general improvement areas, suggesting that while student proficiency in these steps was limited, their impact on overall evaluation was modest. Notably, items in the fourth quadrant, which demonstrated both low achievement and strong association with the comprehensive score, were designated as priority improvement areas. These represent key instructional targets, where enhancing student performance is expected to yield a significant improvement in overall task execution.

In both groups, several procedural items located in the fourth quadrant highlighted key challenges in skill acquisition. These items included performing the aseptic filling process in the correct order, inserting the needle vertically into the vial without approaching from above, and confirming drug dissolution, suggesting that difficulties in these critical steps were linked to lower overall performance regardless of instructional method.

Quadrant-specific differences also underscore the instructional characteristics of each modality. The VR group demonstrated stronger performance in steps requiring spatial coordination, procedural fluidity, and real-time judgment—skills likely supported by the immersive and visual nature of the VR environment. In contrast, the Tx group performed better in steps involving observation and verification, such as fluid measurement, solution mixing, and visual inspection for contaminants. These results imply that text-based materials may better support tasks grounded in rule-following and careful confirmation.

It should be noted, however, that achieving perfect equivalence between VR-based and text-based learning materials across different modalities is inherently challenging. Although both the VR application and the text-based materials comprehensively covered all evaluation items and procedural steps, the text-based materials in this study were designed without visual elements to represent a conventional minimal self-study format. Therefore, the presence or absence of visual and interactive components may have influenced learning outcomes and potentially confounded the comparison between modalities. Future studies incorporating intermediate comparators, such as illustrated manuals or video-based learning, may help to further clarify modality-specific effects.

The items that appeared exclusively in the fourth quadrant further highlighted method-specific challenges. In the VR group, lower achievement in tasks such as discarding the vial and dissolving the drug properly may have resulted from an overemphasis on dynamic procedural flow, leading students to overlook more subtle but essential steps. In the Tx group, poor performance on tasks involving negative pressure techniques and needle insertion angles likely stemmed from the absence of spatial or kinetic information in the materials. These observations suggest that VR and text-based approaches each emphasize different aspects of procedural learning and that combining them could enhance the overall effectiveness of aseptic technique education.

These findings suggest that VR-based learning materials may provide educational benefits comparable to conventional text-based learning, particularly in terms of procedural efficiency. Moreover, the VR application appears particularly effective in enhancing procedural efficiency by enabling learners to comprehend the overall structure and sequence of operations in a clear and organized manner. A previous study examining the use of an educational VR application for objective structured clinical examinations (OSCEs) in medical education also demonstrated superior educational outcomes compared to a control group. Furthermore, it supported the application of VR-based tools in assessing clinical competence, consistent with the findings of the present study [16, 17].

Several limitations were identified during the study. First, this was a single-center pilot study of 17 subjects; thus, it is considered that further validation is needed for generalization. The usefulness of VR needs to be tested with more subjects in the future. Second, VR technology in pharmacy education is still in its infancy, with non-standardized content and assessment processes. Third, VR technology has limited capabilities with respect to practical skills, and VR may not fully replicate the complexities of actual pharmacy practice. Finally, accessibility may be an issue for students with disabilities or other health conditions (such as motion sickness or dizziness). While taking these issues into consideration, the use of VR technology will enable the reproduction of realistic clinical environments and repetitive learning, which have been difficult with traditional educational methods, and will greatly contribute to the enhancement of practical skills learning. Further development of pharmacy education incorporating VR is expected in the future. Students had reached a level at which they were able to perform aseptic procedures under the instructor’s guidance at the completion of the prior hands-on training session. However, in the absence of objective baseline proficiency data and given the small sample size, random allocation alone may not have fully balanced individual differences in manual dexterity or prior learning outcomes, which could have acted as potential confounders. The frequency and extent of independent review during the 2–3 month interval were not recorded; therefore, differences in background preparation between groups cannot be excluded. As evaluations were conducted by a single assessor, inter-rater reliability could not be assessed. Although this approach was adopted to reduce variability in this pilot study, the inability to examine inter-rater reliability represents a study limitation.

## Conclusions

The results of this study suggest that even in aseptic preparation training, which requires more complex procedures than dispensing oral medications, the VR application-based learning method may be more effective than traditional text-based learning in helping students acquire procedural workflows and improve operational fluency. This practical pharmacy education approach using a VR application allows students to progress through each step by performing the actions themselves, which may facilitate learning procedural workflows compared with traditional text-based learning. In the future, it will be essential to conduct additional studies with a larger number of students to verify the reproducibility and reliability of these findings.

## Data Availability

The datasets generated and/or analyzed during the current study are available from the corresponding author on reasonable request. The VR application used in this study is commercially available as the “Pharmacy Pre-Learning VR Training Service” provided by FUJIFILM System Services Co., Ltd.

## Abbreviations

VR: Virtual Reality
Tx: Textbook-based learning
